# Intracellular complement (complosome) is expressed in hematopoietic stem/progenitor cells (HSPCs) and regulates cell trafficking, metabolism and proliferation in an intracrine Nlrp3 inflammasome-dependent manner

**DOI:** 10.1038/s41375-023-01894-0

**Published:** 2023-04-13

**Authors:** Mariusz Z. Ratajczak, Mateusz Adamiak, Ahmed Abdelbaset-Ismail, Kamila Bujko, Arjun Thapa, Vira Chumak, Stephanie Franczak, Katarzyna Brzezniakiewicz-Janus, Janina Ratajczak, Magdalena Kucia

**Affiliations:** 1 Stem Cell Institute at James Graham Brown Cancer Center, University of Louisville, Louisville, KY USA; 2 Department of Regenerative Medicine Warsaw Medical University, Warsaw, Poland; 3 Department of Hematology, University of Zielona Gora, Hospital Gorzow Wlkp., Zielona Góra, Poland

**Keywords:** Haematopoietic stem cells, Stem cells

## To the Editor:

For many years it was envisioned that components of the complement cascade (ComC), including C3 and C5 proteins, are exclusively synthesized in the liver. However, novel data indicates that they are also expressed by normal lymphocytes and, as reported, activate intracellular receptors in an intracrine-dependent manner [1]. This novel regulatory loop operating in lymphocytes has been described as ‘’complosome” and orchestrates T cell responses and metabolism [2]. To explain this intriguing data, C3 initially appeared in ancient single-cell organisms and was involved in the regulation of metabolism [1]. With time, it underwent evolution into “non-canonical” C3 that, as an intracellular protein, retains some metabolic activities and into “canonical” C3 involved in immune functions as a guardian of pathogen detection and removal [1].

For over 20 years, our team has studied the interaction of the immune system with HSPCs; we have demonstrated that ComC, as a soluble arm of innate immunity, regulates migration, mobilization, homing and engraftment of normal hematopoietic stem progenitor cells (HSPCs) [3]. We have also demonstrated that C3-KO and C5-KO mice are poor mobilizers [4–7] of HSPCs and engraft poorly with transplanted wild type (WT) bone marrow mononuclear cells (BMMNCs) [4–7]. This data implicates involvement of liver derived as well as potentially intracellular complement.

Therefore, based on the fact that hematopoiesis and lymphopoiesis have a common hemato/lymphopoietic stem cell origin and that similar mechanisms regulate the biology of these cells [3, 8], we asked if complosome is also expressed and functional in normal HSPCs. We also asked if intracellular ComC could activate the intracellular innate immunity pattern recognition receptor (PRR) known as the Nlrp3 inflammasome that, as we demonstrated, is also expressed by HSPCs and regulates trafficking, metabolism and proliferation of these cells [9].

**Fig. 1:**
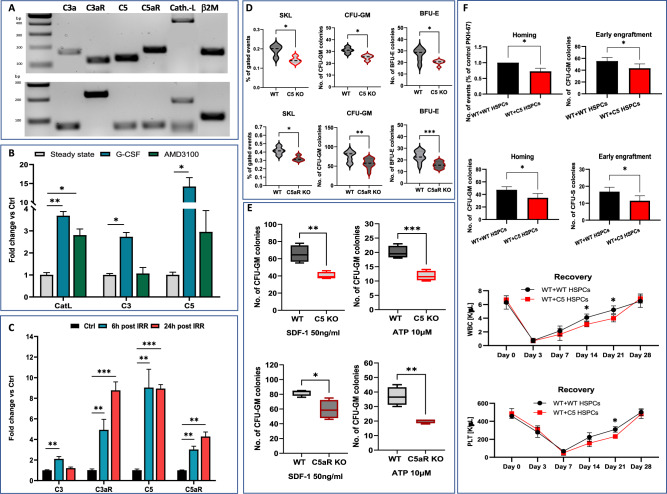
Intracellular complement (complosome) regulates trafficking of HSPCs. Expression of mRNA for complosome elements in human CD34 + CD38- (upper panel) and murine (lower panel) SKL cells. Representative PCR data is shown (**A**). Upregulation of mRNA for complosome elements in PB MNC after mobilization by G-CSF (3 doses; 150 µg/kg) or AMD3100 (1 dose, 5 mg/kg) (**B**). Changes in expression of complosome mRNA in isolated non-hematopoietic cells from mice myeloablated for transplantation by irradiation (1000 cGy) (**C**). Decrease in number of SKL cells and clonogeneic CF-GM and BFU-E in BM from C5-KO (upper lanel) and C5aR-KO (lower panel) mice (**D**). Clonogeneic progenitors (CFU-GM) isolated from C5-KO (upper panel) and C5aR-KO (lower panel) mice show impaired chemotaxis to SDF-1 and ATP gradients (**E**). C5-KO BMMNCs were transplanted along with normal control WT BMMNCs into WT recipients. Left panel shows decreased 24- hour homing of transplanted BMMNCs as assayed by enumeration of PKH-67 labeled cells and the number of CFU-GM progenitors in the BM of transplanted mice. Right panel—number of CFU-GM progenitors in BM and CFU-S in spleens of transplanted mice at day 12 after injection of BMMNC. Lower panel—recovery of WBC and platelets (PLT) in PB of transplanted animals (**F**). The data are presented as means ± SE, and an unpaired Student’s *t*-test was used for the determination of significance (*p ≤ 0.05, **p ≤ 0.01, ***p ≤ 0.001).

To address a potential role of complosome in normal hematopoiesis, we first isolated from murine bone marrow (BM) Sca-1^+^c-kit^+^lin^-^ (SKL) cells and evaluated by RT-PCR expression of mRNA for crucial complosome components. Figure 1A shows the presence of mRNA for C3, C3aR, C5, C5aR, and C3 cathepsin-L (CatL) involved in cleavage of C3 and intracellular activation of ComC [1, 2]. Next, we compared expression of CatL, C3 and C5 between peripheral blood MNCs (PBMNCs) and BMMNC and noticed significantly higher expression, in particular mRNA for C5, Supplementary Fig. 1A. To explain our previous data demonstrating that C3-KO and C5-KO mice are poor mobilizers [5, 6], and to elucidate potential involvement of complosome, we evaluated levels of complosome components in PBMNCs from wild type (WT) mice in steady state conditions and in G-CSF- and AMD3100-mobilized animals. We noticed intracellular upregulation of mRNA for CatL, C3 and C5 in PBMNC after mobilization (Fig. 1B). Again, to explain impaired homing and engraftment of WT BMMNC in C3-KO [4] and C5-KO [7] mice, we asked if complosome components are upregulated in BM non-hematopoietic cells after myeloablative conditioning for transplantation. To address this question, WT mice were lethally irradiated by 1000 cGy. We then measured levels of mRNA for C3, C3aR, C5 and C5aR in BM non-hematopoietic CD45R (B220), TCRγδ^-^ and TCRβ^-^ cells (Fig. 1C). Again, upregulation in expression of mRNA for these complosome components indicates a potential role of intracellular complosome in homing and engraftment of transplanted cells.

Liver derived ComC becomes activated in zymogen-type steps, where C3 is cleaved/activated in response to classical-, mannan lectin- and alternative- complement activation pathways [8]. Activation of C3 leads subsequently to formation of C5 convertase that cleaves C5 to C5a, the most potent mediator of the distal executive pathway of ComC activation [8]. Our previously published data revealed normal hematopoietic parameters in C3-KO mice and engraftment potential of C3-KO BMMNC in WT animals [4]. To learn more about a potential role of complosome, we focused on the distal executive pathway of ComC activation and employed C5-KO and C5aR-KO mice as models. We hypothesized that, in mice, intracrine C5a-C5aR signaling could regulate a pool of HSPCs and their trafficking.

First, we noticed that C5-KO and C5aR-KO mice had a significantly reduced (~15–20%) number of SKL cells as well as clonogenic CFU-GM and BFU-E in BM (Fig. 1D). In Transwell chemotaxis studies, these cells also showed defective migration (by ~30–50%) to stromal derived factor-1 (SDF-1), the main BM homing chemoattractant, as well as to extracellular adenosine triphosphate (eATP), a supportive homing factor (Fig. 1E). Next, we performed in vivo cell homing and engraftment studies with BMMNCs isolated from C5-KO mice transplanted into WT recipients. Figure 1F shows that the number of transplanted PKH67-labeled cells as well as donor-derived clonogenic progenitors was reduced 24 h after transplantation into lethally- irradiated normal recipients’ BM as compared to control WT cells. Similarly, we observed 12 days after transplantation a decrease in the number of C5-KO cells-derived colony forming units in the spleen (CFU-S) and CFU-GM in BM in transplanted WT recipients as compared to transplanted WT cells. Moreover, we observed delayed recovery of peripheral blood counts after transplantation of C5-KO cells in WT recipients (Fig. 1F). In parallel, we observed similar differences for C5aR-KO cells transplanted to WT mice as well as WT cells transplanted to C5aR-KO recipients (Supplementary Fig. 2A). Moreover, C5-KO mice displayed a significant defect in hematopoietic recovery after sublethal (650 cGy) irradiation as compared to their normal littermates (Supplementary Fig. 1B). As mentioned, C5-KO mice engraft poorly with WT BMMNC [7]. To address a potential role of C5aR, we transplanted C5aR-KO animals with WT BMMNC and similarly noticed a defect in engraftment. (Supplementary Fig. 2B). This data corroborated complosome activation in the BM microenvironment after conditioning for transplantation (Fig. 1C).

**Fig. 2:**
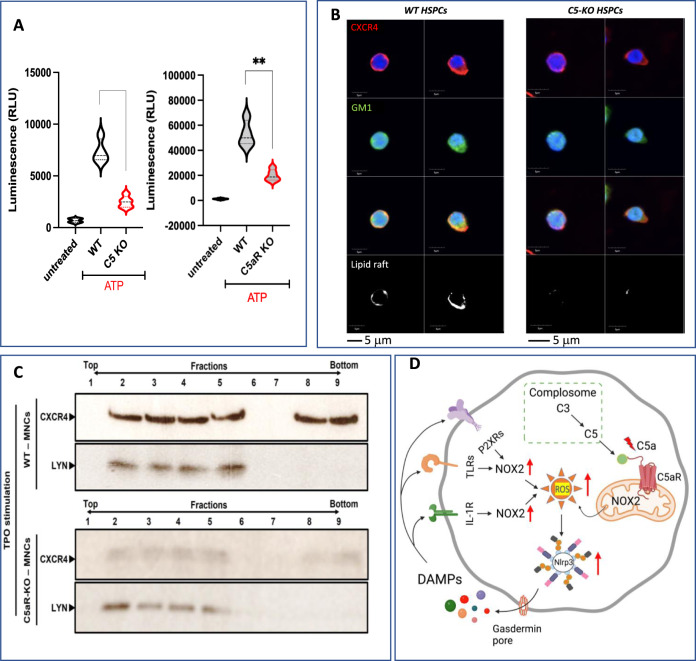
Defect of Nox2-ROS-Nlrp3 inflammasome axis in C5-KO mice. Impaired ATP-mediated activation of Nlrp3 inflammasome in SKL cells from C5-KO and C5aR-KO mice. Nlrp3 inflammasome activation was evaluated by Caspase-Glo® 1 Inflammasome assay (Promega). Experiments were repeated three times The data are presented as means ± SE, and an unpaired Student’s *t*-test was used for the determination of significance (**p ≤ 0.01). (**A**). Defective lipid raft formation in SKL cells from C5-KO mice. Representative images of cells stimulated with SDF-1 (50 ng/ml) and LL-37 (2.5 μg/ml), stained with cholera toxin subunit B (a lipid raft marker), conjugated with FITC and rat anti-mouse CXCR4 followed by anti-rat Alexa Fluor 594, and evaluated by confocal microscopy for formation of membrane lipid rafts. Lipid rafts were formed in SKL cells from WT but not in SKL cells isolated from C5-KO mice (**B**). Western blot analysis of CXCR4 incorporation into MLRs within various fractions of cell membranes. The membranes enriched in lipid rafts are noted in fractions 2˗5. Murine mononuclear cells (MNCs) were cultured with [KL + IL-3+TPO] in serum-free medium for 1 hour at 37 °C. MNCs cultured only in serum-free medium served as a control. CXCR4 expression was evaluated in the membrane fractions isolated from lysates of unstimulated (upper panel) and stimulated cells (lower panel) by western blot. Lyn has been employed as a marker of lipid rafts. (**C**). We postulate complosome generates, in a Nox-2-dependent manner, ROS in response to stimulation of C5aR expressed on the outer cell membrane by intracellular C5a. Subsequently, ROS generated in the cytosol activate the Nlrp3 inflammasome which releases several DAMPs, or alarmin, including IL-1β, S100A8/A9, HMGB-1, and eATP. These DAMPs then interact with their corresponding receptors on the cell surface to produce more ROS, further augmenting Nlrp3 inflammasome activation. These signals, within a non-toxic, “hormetic zone of activation,” enhance metabolism and migration of HSPCs (**D**). (Scheme shown in **D**—Partially modified from our review (10.1007/s12015-023-10533-1) licensed under a Creative Commons Attribution 4.0 International License, http://creativecommons.org/licenses/by/4.0/).

Our previously published data established a link between HSPC migration and activation of the Nlrp3 inflammasome [10]. Since C5-KO and C5aR-KO cells performed poorly in Transwell migration assays and in vivo homing/transplantation studies, we evaluated activation of the Nlrp3 inflammasome in mutant cells after stimulation with its potent activator, eATP. Figure 2A shows, as expected, a defect in Nlrp3 inflammasome activation in murine BM cells from C5- and C5aR-deficient mice. Of note, the Transwell migration of C5-KO cells and mobilization in C5-KO mice was restored after activation of Nlrp3 inflammasome by nigericine (not shown).

In our recent paper, we also demonstrated that the Nlrp3 inflammasome stimulates HSPC migration and metabolism by stimulating cholesterol and sphingolipid synthesis for the assembly of membrane lipid rafts (MLRs) [11]. These lipid structures float freely in the membrane bilayer and play an important role in assembling cytosolic signaling molecules and surface receptors for growth factors, cytokines, chemokines bioactive lipids, extracellular signaling nucleotides, adhesion molecules and cell surface expressed enzymes, which together regulate several cell pathways [12, 13]. Therefore, MLRs behave as some type of sorting hub for cell surface “*raftophilic*” receptors and play a role in their optimal signaling. We asked if a defect in MLRs formation could explain a defect in the migration and homing of HSPCs from C5-KO and C5aR-KO mice.

We evaluated MLRs formation after cell stimulation by MLRs-promoting LL-37 peptide by i) confocal microscope and ii) Western blot analysis of outer cell membrane fractions. SKL cells from C5-KO mice showed, upon stimulation, defective MLRs formation (Fig. 2B, C). Next, we stimulated SKL cells from C5-KO and WT mice with a cocktail of hematopoietic growth factors (KL + IL-3+TpO), and analyzed mRNA expression for key enzymes involved in the synthesis of MLR lipid components, including cholesterol, sterol regulatory element-binding protein 2 (SREBP2), 3-hydroxy-3-methyl-glutaryl-coenzyme A reductase (HMGCR), hydroxymethylglutaryl-CoA synthase (HMGCs), and acid sphingomyelinase (ASMAse). We observed a decrease in expression of these enzymes in C5- and C5aR- deficient cells (Supplementary Fig. 3). Moreover, we also noticed decreased expression of crucial enzymes involved in glycolysis: Glucokinase **(**GK), Glucose transporter 2 **(**GLUT2), Phosphofructokinase (PFKFB3), Glucose-6-phosphate dehydrogenase **(**G6PD), and large neutral amino acid transporter small subunit 1 (LAT1), a transmembrane amino acid transporter (Supplementary Fig. 3). This data indicates a defect in the activation of metabolic pathways in C5-KO and C5aR-KO stimulated cells.

In our previous work, we explained metabolic effects observed in stimulated HSPCs as a result of nitric oxide synthetase-2 (Nox2) activation [11]. This activation is a primary source of reactive oxygen species (ROS) which regulate the intracellular redox state and thus modulate the proliferation and migration of HSPCs [14]. For many decades, ROS have been considered cellular waste products that can lead to oxidation events and permanent damage to DNA, lipids, and proteins. Recently, depending on activation level ROS are also recognized as “signaling molecules” having nuanced roles in regulating almost all aspects of cell function [14]. Evidence has accumulated that ROS, at a safe, beneficial for cells, “hormetic” level of activation, play an important role as signaling molecules that modify the activity of numerous enzymes through oxidation of cysteine and methionine residues. The most essential ROS is hydrogen peroxide (H_2_O_2_), which reversibly oxidizes redox-sensitive cysteine and methionine residues buried within transcription factors, enzymes, and structural proteins [14]. Therefore, ROS-mediated “redox signaling” controls cell function by modifying the expression and activity of several metabolic enzymes and transcription factors [14].

Since C5aR, as a part of complosome, is expressed on mitochondria membrane [1, 2], stimulation of mitochondrial C5aR by C5a promotes release of ROS that directly activates the Nlrp3 inflammasome (Fig. 2D). Subsequently, activated Nlrp3 inflammasome triggers release of several danger associated molecular pattern (DAMPs) mediators, or alarmins, from the cells in an autocrine manner. Next, these mediators activate their specific receptors expressed on the cell surface. Since these receptors are associated with Nox2, as a consequence intracellular ROS additionally increase and potentiate ROS signaling-dependent metabolic effects [11, 14, 15].

We conclude, that complosome is a novel regulator of hematopoiesis via intracrine activation of Nox2-ROS-Nlrp3 inflammasome. Impaired intracellular Nox2-ROS signaling in C5-KO mice HSPC s explains the defect in the supply of MLR lipid components crucial for MLRs assemble and optimal “raftophilic” receptors signaling. On the other hand, a decrease in glucose and amino acid metabolism has additional negative effects on HSPC metabolism, migration and proliferation. This reported herein complosome co-regulated axis may become a therapeutic target to optimize trafficking and metabolism of normal HSPCs.

## Supplementary information

Legends for Supplementary Figures

Supplementary Figure 1

Supplementary Figure 2

Supplementary Figure 3

## Data Availability

Detailed data available upon request.
