# Supplementary material for: Intracellular complement (complosome) is expressed in hematopoietic stem/progenitor cells (HSPCs) and regulates cell trafficking, metabolism and proliferation in an intracrine Nlrp3 inflammasome-dependent manner
Source: Leukemia. 2023 Apr 13;37(6):1401–5. doi: 10.1038/s41375-023-01894-0 (PMC10244163; doi:10.1038/s41375-023-01894-0)
Supplement: Supplementary file 1 — Legends for Supplementary Figures [file 41375_2023_1894_MOESM1_ESM.docx]

**Legends for Supplementary Figures.**

**Supplementary Figure 1**. **Panel A**. Expression of complosome mRNA by RQ-PCR in PB and BM MNC (n=3). **Panel B**. Recovery of WBC and PLT after sublethal irradiation of C5-KO mice (6 animals per group, *p ≤ 0.01, ****p ≤ 0.0001).

**Supplementary Figure 2**. C5aR-KO cells were transplanted into WT recipients (**A**), or WT BMMNC were transplanted into C5aR-KO recipients (**B**), or WT BMMNC were transplanted into C-KO mice (**C**). Left panels shows decreased 24-hour homing of transplanted BMMNCs as assayed by enumeration of PKH-67 labeled cells and the number of CFU-GM progenitors in the BM of transplanted mice. Middle panels – number of CFU-GM progenitors in BM and CFU-S in spleens of transplanted mice at day 11 after injection of BMMNC. Right panels – recovery of WBC and platelets (PLT) in PB of transplanted animals. (6 animals per group, *p ≤ 0.01).

**Supplementary Figure 3**. Expression of the key enzymes involved in cholesterol synthesis [SREBP2, HMGCs, HMGCR, and ASMAse], glycolysis [GK, GLUT2, PFKFB3 and G6PD] and protein synthesis [SLC7A5/LAT1] were evaluated by qRT-PCR in mRNA samples purified from C5-KO (left panel) and C5aR-KO (right panel) SKL cells in steady state conditions or cultured with [KL+IL-3+TPO] in a serum-free medium for 1 hour at 37 °C. For each experiment, β2-microglobulin was used as an endogenous control. Samples containing only water instead of cDNA were also used per each run as a negative control. (6 animals per group, *p ≤ 0.01).
