# Supplementary material for: Intracellular complement (complosome) is expressed in hematopoietic stem/progenitor cells (HSPCs) and regulates cell trafficking, metabolism and proliferation in an intracrine Nlrp3 inflammasome-dependent manner
Source: Leukemia. 2023 Apr 13;37(6):1401–5. doi: 10.1038/s41375-023-01894-0 (PMC10244163; doi:10.1038/s41375-023-01894-0)
Supplement: Supplementary file 2 — Supplementary Figure 1 [file 41375_2023_1894_MOESM2_ESM.pdf]

A

Expression of complement mRNA in BMMNCs

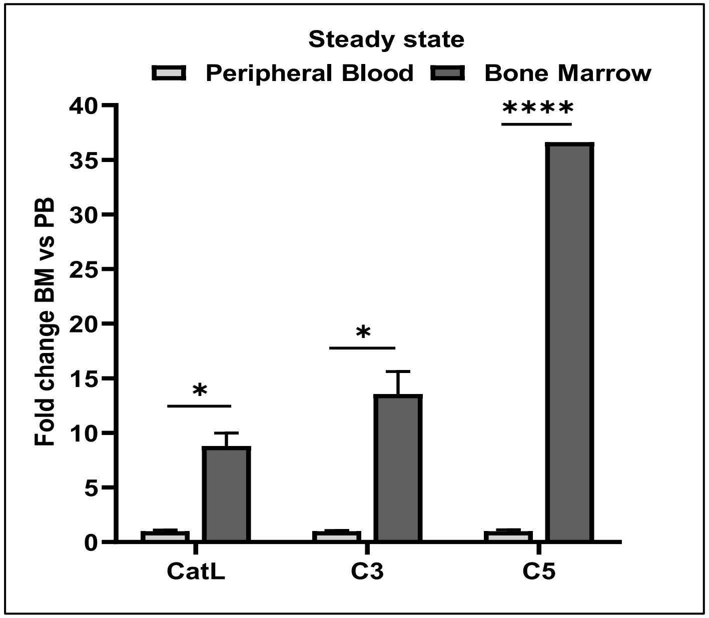

B

C5-KO mice have impaired hematopoietic recovery (WBC andPLTs counts) after sublethal (650 cGy) irradiation

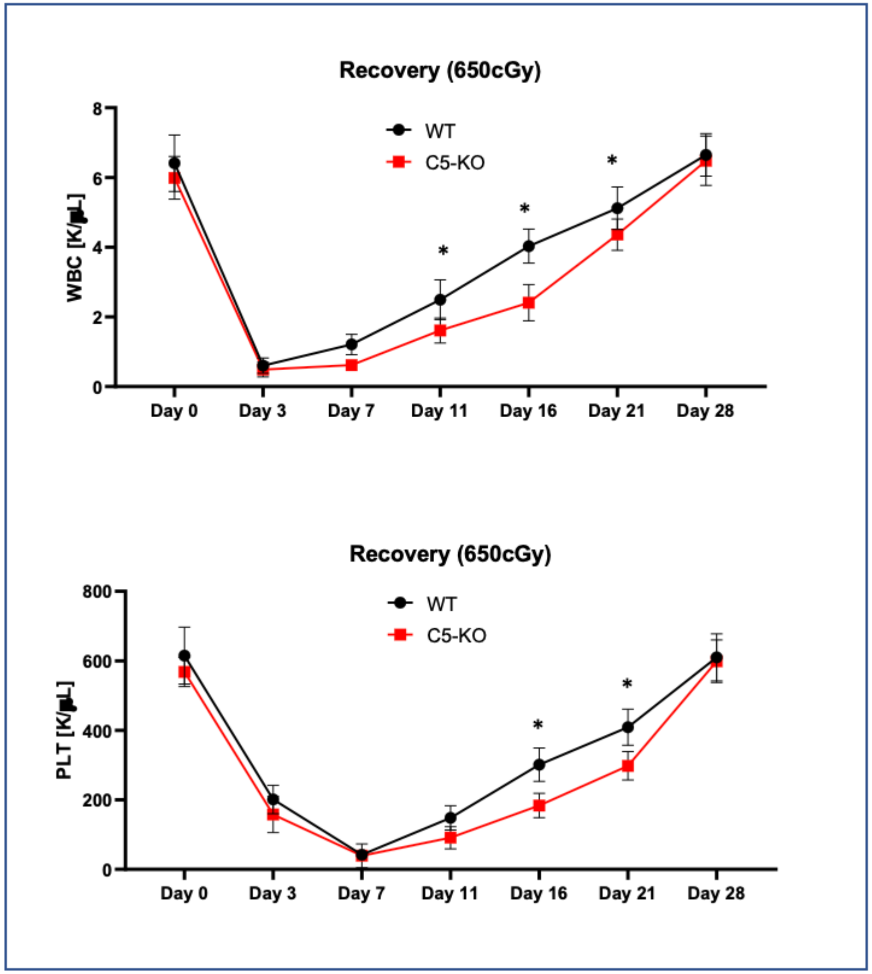

Supplementary Figure 1
