# Supplementary figures and images for: Intracellular complement (complosome) is expressed in hematopoietic stem/progenitor cells (HSPCs) and regulates cell trafficking, metabolism and proliferation in an intracrine Nlrp3 inflammasome-dependent manner
Source: Leukemia. 2023 Apr 13;37(6):1401–5. doi: 10.1038/s41375-023-01894-0 (PMC10244163; doi:10.1038/s41375-023-01894-0)

A

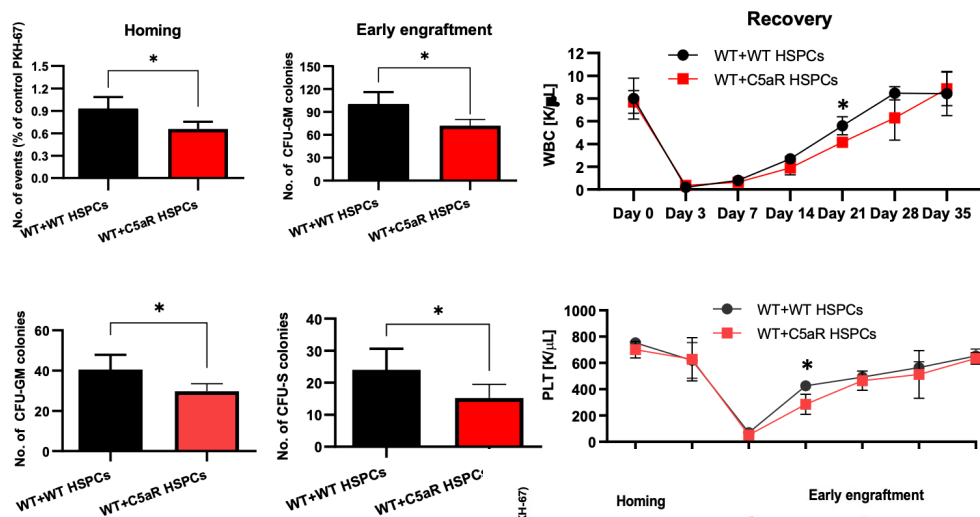

B

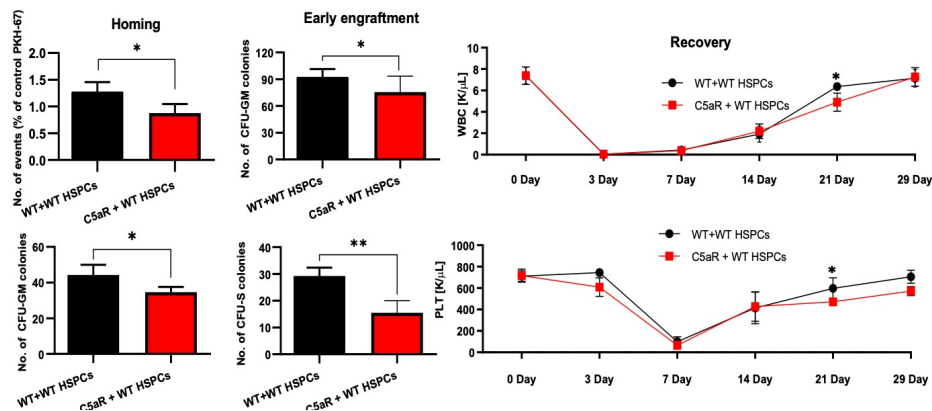

C

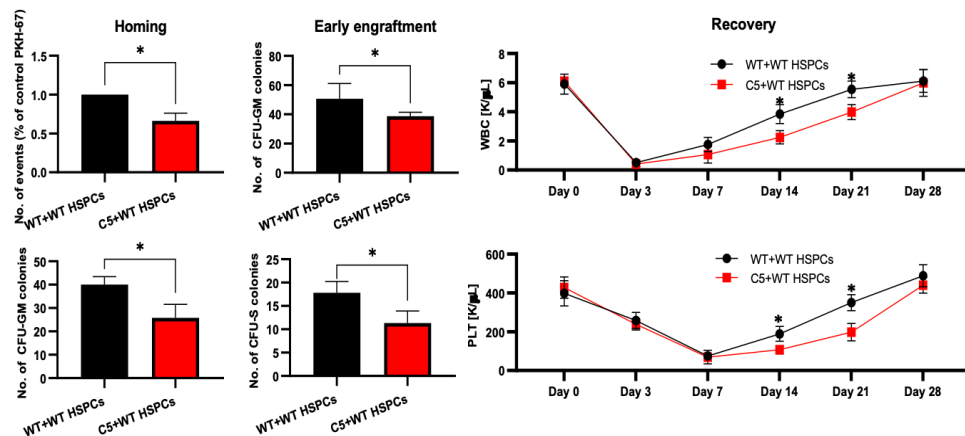

Supplementary Figure 2

Supplement: Supplementary file 3 — Supplementary Figure 2 [file 41375_2023_1894_MOESM3_ESM.pdf]

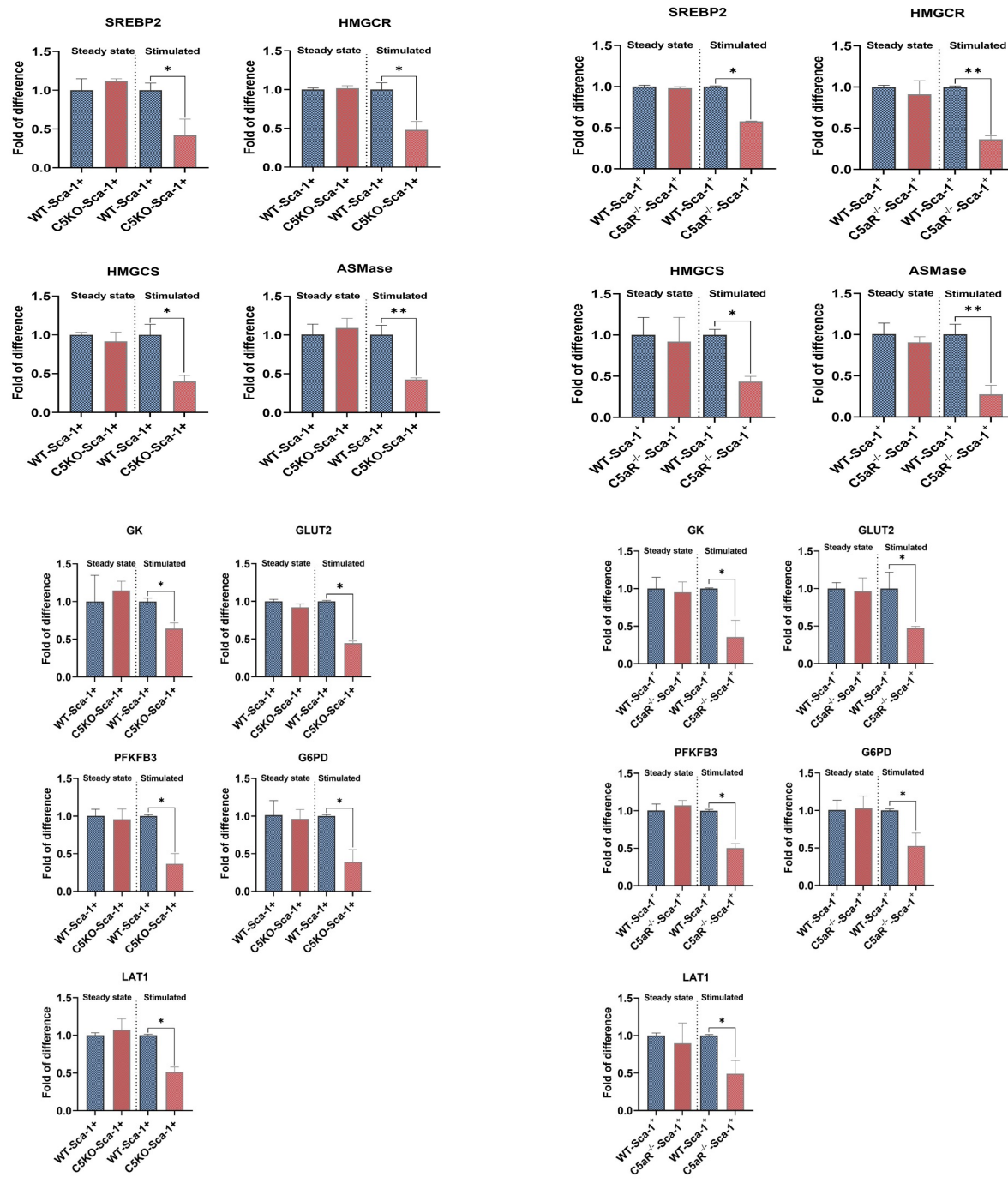

Supplementary Figure 3

Supplement: Supplementary file 4 — Supplementary Figure 3 [file 41375_2023_1894_MOESM4_ESM.pdf]
